# Circulating Interleukin-18 as a Biomarker of Total-Body Radiation Exposure in Mice, Minipigs, and Nonhuman Primates (NHP)

**DOI:** 10.1371/journal.pone.0109249

**Published:** 2014-10-07

**Authors:** Cam T. Ha, Xiang-Hong Li, Dadin Fu, Maria Moroni, Carolyn Fisher, Robert Arnott, Venkataraman Srinivasan, Mang Xiao

**Affiliations:** Radiation Countermeasures Program, Armed Forces Radiobiology Research Institute, Uniformed Services University of the Health Sciences, Bethesda, Maryland, United States of America; ENEA, Italy

## Abstract

We aim to develop a rapid, easy-to-use, inexpensive and accurate radiation dose-assessment assay that tests easily obtained samples (e.g., blood) to triage and track radiological casualties, and to evaluate the radioprotective and therapeutic effects of radiation countermeasures. In the present study, we evaluated the interleukin (IL)-1 family of cytokines, IL-1β, IL-18 and IL-33, as well as their secondary cytokines’ expression and secretion in CD2F1 mouse bone marrow (BM), spleen, thymus and serum in response to γ-radiation from sublethal to lethal doses (5, 7, 8, 9, 10, or 12 Gy) at different time points using the enzyme-linked immune sorbent assay (ELISA), immunoblotting, and cytokine antibody array. Our data identified increases of IL-1β, IL-18, and/or IL-33 in mouse thymus, spleen and BM cells after total-body irradiation (TBI). However, levels of these cytokines varied in different tissues. Interestingly, IL-18 but not IL-1β or IL-33 increased significantly (2.5–24 fold) and stably in mouse serum from day 1 after TBI up to 13 days in a radiation dose-dependent manner. We further confirmed our finding in total-body γ-irradiated nonhuman primates (NHPs) and minipigs, and demonstrated that radiation significantly enhanced IL-18 in serum from NHPs 2–4 days post-irradiation and in minipig plasma 1–3 days post-irradiation. Finally, we compared circulating IL-18 with the well known hematological radiation biomarkers lymphocyte and neutrophil counts in blood of mouse, minipigs and NHPs and demonstrated close correlations between these biomarkers in response to radiation. Our results suggest that the elevated levels of circulating IL-18 after radiation proportionally reflect radiation dose and severity of radiation injury and may be used both as a potential biomarker for triage and also to track casualties after radiological accidents as well as for therapeutic radiation exposure.

## Introduction

Radiation injuries are heterogeneous disorders that involve many pathophysiological pathways and affect both cells directly exposed to radiation and cells not directly exposed. Normal tissue injuries induced by ionizing radiation differ depending on the type of radiation, dose and dose-rate of radiation exposure, and the varied radiation-tolerances in target organs and cells. For example, a γ-radiation dose above 1 Gy in humans or mice poses a risk of destruction of the bone marrow (BM) and damage to the hematopoietic system [1], [2], whereas only high-dose (10 Gy or more) total-body irradiation (TBI) in experimental mice can result in acute generalized gastrointestinal (GI) syndrome with loss of intestinal crypts, damage to crypt stem cells, and breakdown of the GI mucosal barrier, leading to animal death [3]–[5]. In addition, total-body ^60^Co γ-radiation induced 90% mortality within 30 days (LD_90/30_) with a 95% confidence interval (CI) at doses of 9.6 Gy in CD2F1 mice [6], 1.86 Gy in Gottingen minipigs [7] and 7.56 Gy in rhesus macaques (LD_90/60_ without supportive care) [8], showing that the radiation sensitivity in various animal species differs significantly. The mechanisms of these complex biological responses of tissues to harmful radiation damage are not well understood, and rapid, easy-to-use, inexpensive and accurate methods for assessing radiation doses and evaluating radiation-induced injury as well as the effects of radiation countermeasures are not available, although multiple parameter biomarkers have been reported [9]–[11].

Radiation causes cellular DNA damage leading to “danger signals” and antigen release. These signals and antigens are important proinflammatory causal factors involved in proinflammatory and immune reactions in target cells [12], [13]. Massive radiation-induced pro-inflammatory factor release from injured cells may further result in stress response signal activation and cell damage and depletion [14]–[18]. The interleukin (IL)-1 family cytokines are linked closely to the innate immune response and as the first line of host defense against stress-induced acute and chronic inflammation [19], [20]. IL-1 family members IL-1β, IL-18, and IL-33 play key roles in inflammatory and immune responses and have been described as having significant influence on the pathogenesis of diseases [21]. IL-1β and IL-18 are first synthesized as low levels of inactive precursor presenting in healthy human and animal cells, and after cleavage by active caspase-1 become mature active factors secreted in response to disease, stress, and inflammatory stimuli [22], [23]. IL-1β induces production of secondary inflammatory factors IL-6, IL-8, tumor necrosis factor-alpha (TNFα), interferon-gamma (IFNγ), granulocyte colony-stimulating factor (G-CSF) and granulocyte–macrophage colony-stimulating factor (GM-CSF) [24], [25]. IL-18 has a role in destructive inflammatory disorders and stimulates neutrophil migration and activation as well as T helper 1 (Th1) cell differentiation and IL-2, GM-CSF and IFN-γ secretion in a variety of cell types through Toll-like receptor (TLR) signaling [20], [23]. In contrast, full-length IL-33 is bioactive and its inactivation results from caspase-1-mediated cleavage. Bioactive IL-33 exists in cells and the inactive cleavage form is released by necrotic and dead cells. IL-33 can induce release of T helper 2 (Th2) cells, as well as Th2 type cytokines such as IL-4, IL-5 and IL-13 [26], [27]. Ionizing radiation-induced IL-1β, IL-6, IL-8, G-CSF and/or GM-CSF production in mouse BM and intestinal cells and human osteoblast cells have been reported by our laboratory [5], [15], [18]. A recent study demonstrated that whole-body low dose (0.05–2 Gy) radiation induced IL-12 and IL-18 secretion by mouse peritoneal macrophages [13]. Because radiation tolerances vary in different tissues and cells, we hypothesize that elevated levels of circulating IL-1 family cytokines in serum after TBI proportionally reflect the radiation doses and severity of radiation-induced tissue damage, and can be used as potential biomarkers of ionizing radiation injury. In the current study we compared IL-1β, IL-18, and IL-33, as well as their downstream secondary cytokine expression in mouse BM, spleen, thymus, and serum after γ-radiation from sublethal to lethal doses (5, 7, 8, 9, 10, or 12 Gy) at different time points and demonstrated that the most radiosensitive and stabile cytokine is IL-18 in mouse serum. We further evaluated levels of circulating IL-18 in minipig plasma and nonhuman primate (NHP) serum after radiation exposure. Because hematological biomarkers of exposure to ionizing radiation are well characterized and used in medical management of radiological casualties [28], in this study, close correlations were found between the new radiation biomarker IL-18 and well known hematological radiation biomarkers [29] in our animal models. The present study provides a novel method for determining radiation injury by quantitation of circulating IL-18 in different animal species using ELISA (enzyme-linked immune sorbent assay).

## Results

### ^60^Co γ-radiation-induced expression of IL-1β, IL-18, and IL-33 in mouse tissues

Previously we demonstrated that total-body γ-radiation induced 50% and 90% mortality within 30 days (LD_50/30_ and LD_90/30_) in CD2F1 mice that had received 8.5 Gy and 9.6 Gy, respectively [6]. Radiation at 8.75 Gy significantly induced apoptosis and death of mouse hematopoietic cells [30], and doses above 10 Gy of total-body irradiation (TBI) destroyed both hematopoietic and gastrointestinal (GI) cells and degraded the GI mucosal-epithelial barrier, which resulted in bacterial translocation from intestines into the blood [5]. Based on these results, the current study was designed so that CD2F1 mice received ^60^Co γ-radiation exposures at 0 (sham irradiated control), 8, 10 or 12 Gy. Radiation-induced pro-inflammatory cytokine release was first evaluated in different mouse tissues up to 9 days post-irradiation, by which time many mice in the 12 Gy irradiated group were dead. The experiments were stopped at day 14 according to the Institutional Animal Care and Use Committee (IACUC) protocol from the Armed Forces Radiobiology Research Institute (AFRRI).

**Figure 1:**
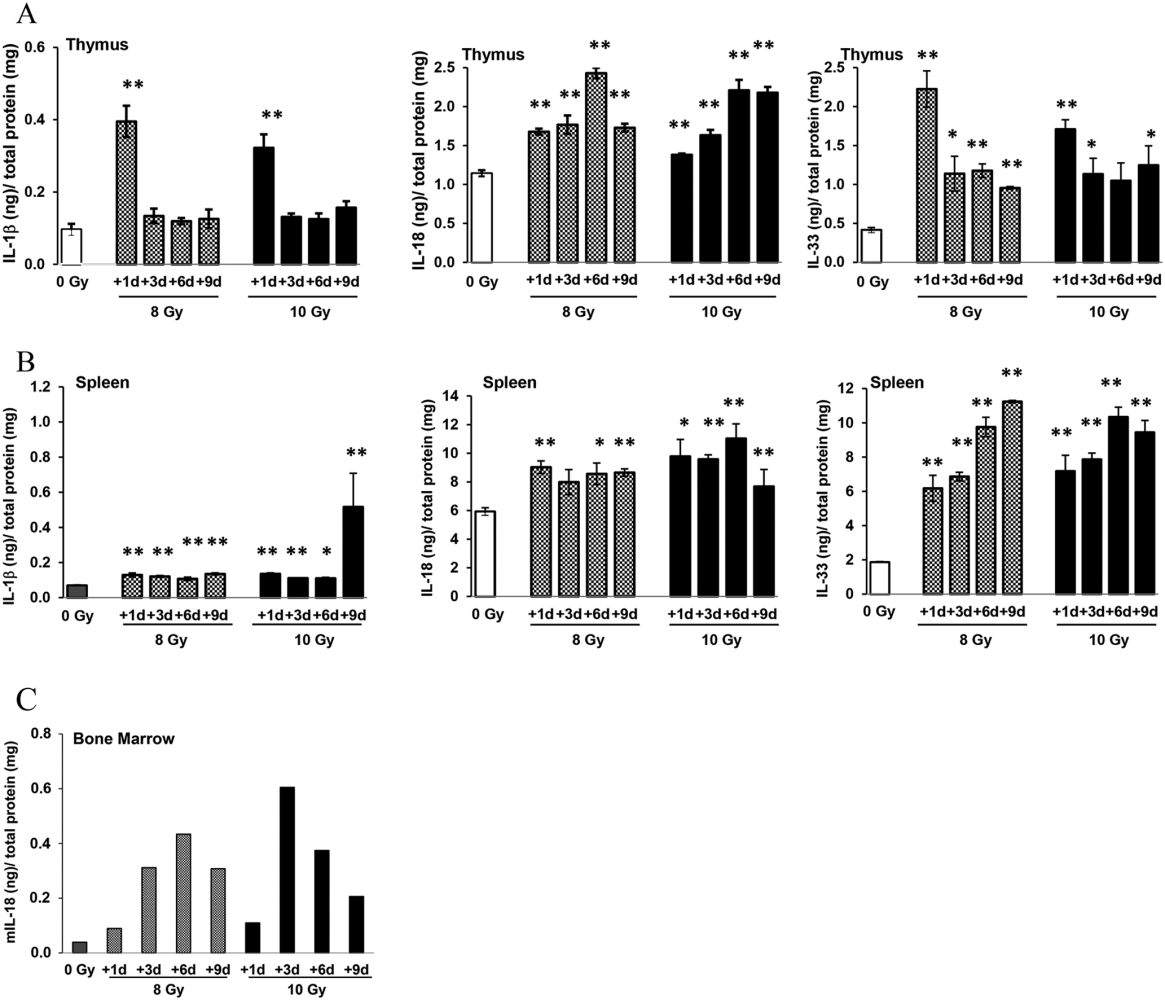
^60^Co-radiation induced expression of IL-1ß, IL-18, and IL-33 in mouse tissues. Mouse thymi, spleens, and BM were collected after 1, 3, 6, and 9 days after 0, 8 and 10 Gy of ^60^Co-TBI. Their homogenates were generated in PBS, and for each assay an equally determined amount of total protein was applied for quantitative detection of IL-1β, IL-18, and IL-33 by ELISA assay. After normalization, the results were displayed as the cytokine levels measured in 1 mg of tissue homogenates. (A) and (B) show the levels of IL-1β, IL-18, and IL-33 in thymus and spleen homogenates, respectively. Results were from a total of three experiments, N = 6/group in each experiment; *p<0.05, **p<0.01; mean ± SD. (C) shows the levels of IL-18 from six BM cell lysates combined and tests were performed in duplicate.

Mouse bone marrow (BM) from femora, humeri, spleens, and thymi were collected on days 1, 3, 6, and 9 after 0, 8, or 10 Gy irradiation (designated as +1 d, +3 d, +6 d, +9 d, with the day of irradiation considered 0 d). Cell homogenates from BM, spleens, and thymi were generated in PBS (phosphate buffered saline). An optimized amount of total protein from each sample in indicated groups (6 mice/group) was applied for determining of IL-1β, IL-18, and IL-33 using quantitative enzyme-linked immune sorbent assay (ELISA). The data are reported as cytokine levels detected in 1 mg of total protein/sample. Results in figure 1A showed that 8 and 10 Gy radiation induced about 4-fold increases of IL-1β and IL-33 in thymi 1 day after irradiation compared to sham-irradiated control. Levels of IL-1β reverted to baseline as shown in sham-irradiated control on day 3 and did not change thereafter, whereas IL-33 expression was significantly higher than control up to 6 (8 Gy) and 9 (10 Gy) days post-irradiation with a peak on day 1. In comparison with IL-1β and IL-33, radiation-induced IL-18 increases were observed on day 1 and reached a peak on day 6, with an approximate 2.5-fold increase compared with 0 Gy. At day 9, IL-18 levels in thymi from the 8 Gy and 10 Gy groups were 1.5-fold (8 Gy) and 2-fold (10 Gy) higher than the sham-irradiated control group, respectively.

We further examined expression of these cytokines in mice spleens (figure 1B). As was the case in thymi, IL-1β expression in spleens was relatively low. However, radiation at 8 and 10 Gy significantly increased its level up to 9 days post-TBI. Baselines of IL-18 and IL-33 were high in spleen cells. A radiation-induced 1.5-fold increase of IL-18 after 8 or 10 Gy irradiation was observed in all samples from day 1 to day 9 post-TBI (p<0.05). In comparison with IL-1β and IL-18, IL-33 increased markedly in spleen samples after irradiation with approximately a 3-fold rise on day 3 and 4- to 5-fold increase on day 6 and 9 post-TBI (p<0.01).

Previously, we reported a transient IL-1β expression in CD2F1 mouse BM in response to γ-radiation [18]. In the current study, we examined IL-18 and IL-33 expression in mouse BM cells after 8 or 10 Gy TBI at different time points. BM samples from each group (6 mice/per group) were pooled for ELISA experiments due to the limited number of BM cells collected from each mouse after irradiation. A 1-fold elevation of IL-18 in mouse BM cells was observed on day 1 after exposure to 8 or 10 Gy, and continually increased up to 5-fold after 8 Gy and 12-fold after 10 Gy 3 days post-TBI. This high level of IL-18 expression lasted up to 9 days post-TBI compared with BM samples from 0 Gy control mice (figure 1C). In contrast, levels of IL-33 in mouse BM were undetectable using the same ELISA method as used in thymus and spleen samples.

**Figure 2:**
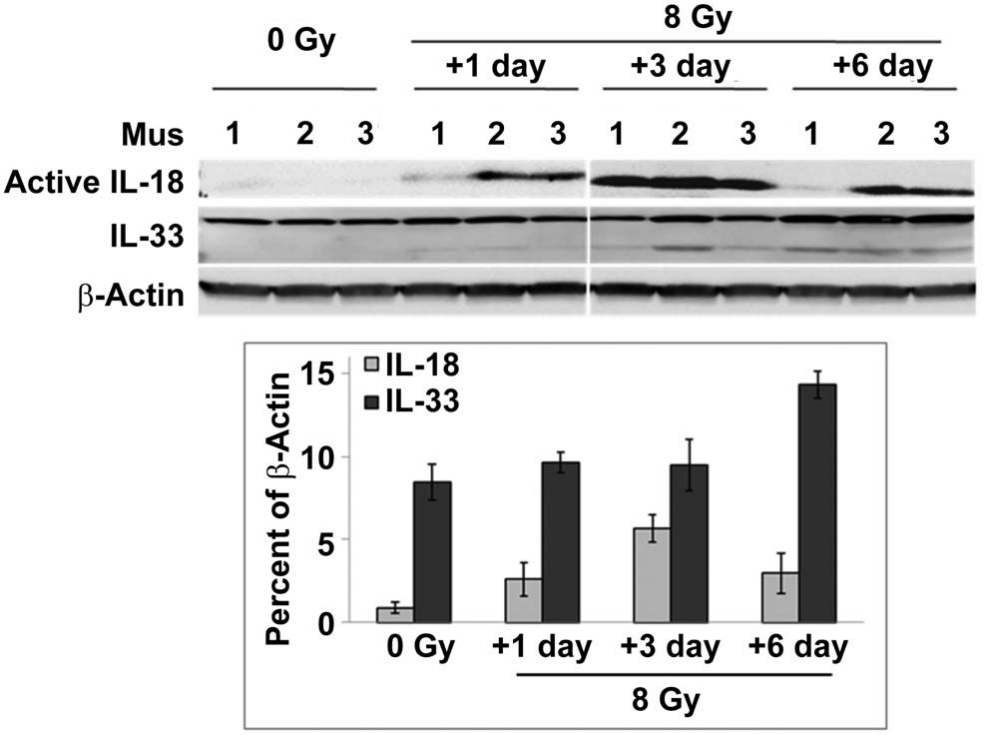
Immunoblotting detection of IL-18 and IL-33 expression in mouse spleen cells after ^60^Co-TBI. Analysis of protein expression of active IL-18 and IL-33 was performed by western blot. SDS-PAGE was conducted with 120 µg of spleen cell homogenates from individual mice, three mice per group. The mature sizes of IL-18 and IL-33 are approximately 18 and 33 KDa, respectively.

Next, to confirm cytokine protein expression in mouse tissues determined by ELISA, immunoblotting with antibodies specific for mouse IL-1β, the active form (18 KDa) of IL-18, and IL-33 were performed in sham-irradiated (0 Gy) and 8 Gy irradiated mouse spleen samples. Figure 2 shows western blot results from one representative of three independent experiments. With spleen samples from 3 mice per group, active IL-18 was expressed on day 1, and significant upregulation occurred on day 3 and day 6 after 8 Gy TBI. Because full-length intracellular IL-33 is bioactive, we further examined full-length IL-33 expression in these spleen cell samples. Radiation-induced IL-33 upregulation was shown 6 days after 8 Gy TBI. IL-1β expression was below the detectable level with the immunoblotting method in spleens. These results were in agreement with results from ELISA experiments.

### ^60^Co γ-radiation increased the secretion of bioactive IL-18 in mouse serum

**Figure 3:**
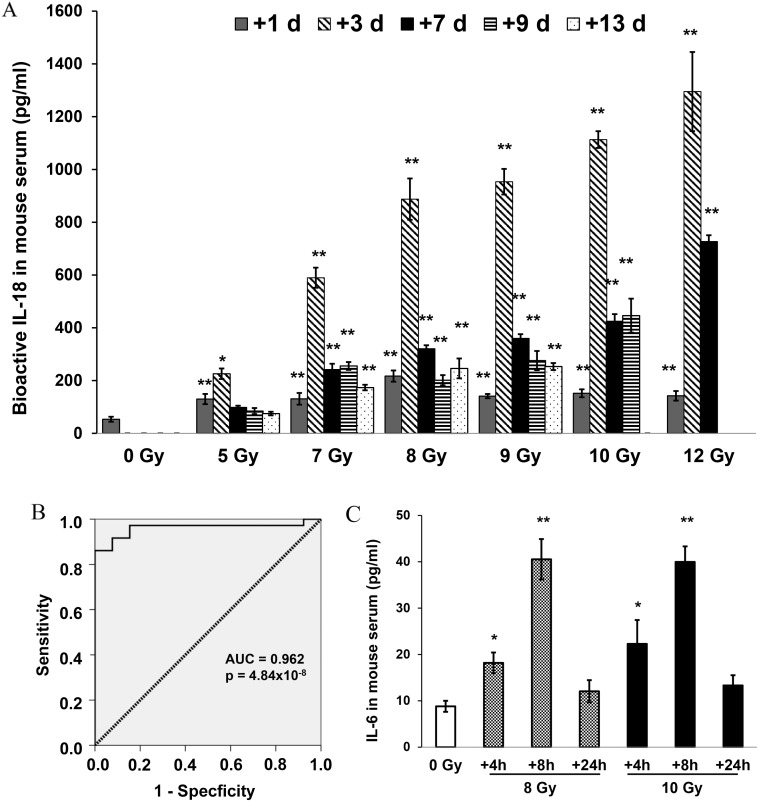
Elevation of IL-18 and IL-6 in mouse serum after ^60^Co-TBI exposure. Mouse sera from (A) 0, 5, 7, 8, 9, 10, or 12 Gy TBI mice were collected 1, 3, 6, 9 and 13 days after irradiation. Serum IL-18 concentrations were determined by ELISA assay, which specifically detects the bioactive form of mouse IL-18. ELISA was conducted using 50–100 µl of serum per individual sample. The values represent the data from four independent experiments (6 mice/group in each experiment, N = 24). *p<0.05, **p<0.01; mean ± SD. (B) ROC curve of serum IL-18 concentration 1 day after gamma-radiation. The area under the curve (AUC) was 0.96 (95% CI 0.91–1.00; P = 4.84×10^−8^). Statistical data of the AUC at 95% CI and AVOVA p-values in comparison of IL-18 concentrations in sham- and 1, 3, 6, 9 and 13 day post-irradiated samples are presented in table 1. (C) Mouse serum from 0 Gy control, and 8, or 10 Gy TBI mice were collected at 4, 8, and 24 h after irradiation. Serum IL-6 concentrations were determined by ELISA using 50–100 µl serum per individual sample. Values represent data from three independent experiments (6 mice/group in each experiment, N = 18). *p<0.05, **p<0.01; mean ± SD.

Results from experiments described above showed different patterns of radiation-induced IL-1β, IL-18 and IL-33 expression in mouse tissues. We next asked whether these cytokines can be determined in mouse serum after radiation exposure. Mouse serum samples from 0 Gy control, and 5, 7, 8, 9, 10, or 12 Gy TBI mice on 1, 3, 6, 9, and 13 days after irradiation were collected and levels of cytokines in serum of individual mice were measured by ELISA. Data from four independent experiments (6 mice/group in each experiment, N = 24) consistently showed that bioactive levels of IL-18 in mouse sera increased significantly after TBI exposure. The concentration of bioactive IL-18 in sera from the unirradiated control group was 53.5±9.4 pg/ml. Its levels were increased significantly from day 1 to day 6 after TBI, reaching a peak at day 3 from 225.9±9.9 pg/ml (5 Gy) up to 1285.2±149.9 pg/ml (12 Gy) (figure 3A). Nine days after irradiation, levels of IL-18 remained higher for 5–10 Gy groups compared with unirradiated control. There was no IL-18 data on day 13 after 10 Gy and on day 9 and 13 after 12 Gy irradiation because most mice did not survive in those groups. In addition, the sensitivity and specificity of IL-18 expression in serum after all doses of γ-radiation at indicated time points were analyzed by receiver operator characteristic (ROC) curves [31]. Figure 3B shows the ROC curve of IL-18 levels in comparison of sham-irradiated vs. 1 day after γ-irradiated samples. The ROC curve analysis is summarized by area under the curve (AUC) at 95% confidence intervals (CI), and *p*-values from statistical analysis (ANOVA) as shown in table 1. With AUC was above 0.94 and p<0.001 in all time points that has been tested, the high specificity and sensitivity of IL-18 in response to radiation was observed after TBI. It is noted that serum samples undergoing several freeze and thaw cycles produced almost identical readings in this ELISA method. In comparison with IL-18, the increased secretions of IL-1β and IL-33, as well as IL-1β’s downstream cytokines IL-6 and IL-8 in mouse sera from irradiated mice were not observed from day 1 to day 9 post-irradiation.

**Table 1:** Performance evaluation of circulating IL-18 as a radiation biomarker in mouse serum.

Time post-irradiation	0 Gy vs. radiation	Data
**+1 day**	*P*-value	4.84×10^−8^
	AUC	0.962
	95% CI	0.912–1.000
**+3 day**	*P*-value	2.15×10^–11^
	AUC	1.000
	95% CI	1.000–1.000
**+6 day**	*P*-value	3.26×10^−7^
	AUC	0.983
	95% CI	0.956–1.000
**+9 day**	*P*-value	1.4×10^−5^
	AUC	0.955
	95% CI	0.904–1.000
**+13 day**	*P*-value	8.09×10^−7^
	AUC	0.94
	95% CI	0.876–1.000

Data were analyzed using IBM-SPSS program (SPSS Statistics Professional). A rough guide for assessing the utility of a biomarker based on its AUC is as follows: 0.9–1.0 = excellent; 0.8–0.9 = good; 0.7–0.8 = fair; 0.6–0.7 = poor; 0.5–0.6 = fail [31].

ANOVA *p*-value and ROC analysis AUC at 95% CI: 0 Gy vs. all radiation doses (5, 7, 8, 9, 10, and 12 Gy).

We further extended the experiments and examined IL-1β, IL-6, IL-8, IL-18 and IL-33 expression in mouse serum at early time points (4 and 8 h) after irradiation. Our results demonstrated that radiation-induced elevation of IL-6 in mouse serum was observed as early as 4 h and reached a peak at 8 h before returning to the baseline level as shown in non-irradiated control samples at 24 h post-irradiation (figure 3C). In contrast, radiation-induced elevation of IL-1β, IL-8, IL-18, and IL-33 were not shown by ELISA in mouse serum at the early time points after irradiation.

**Figure 4:**
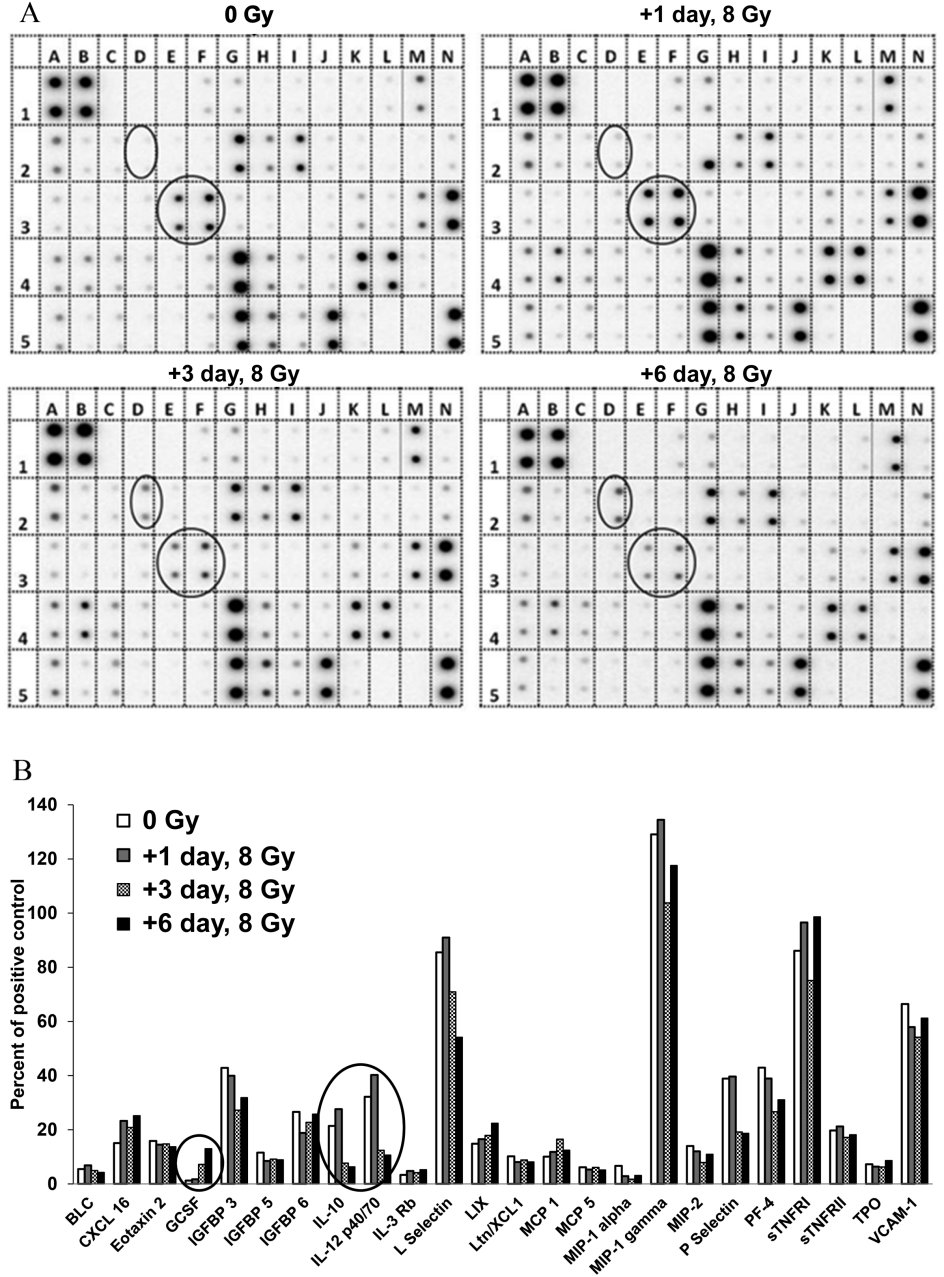
Cytokine antibody array in mouse serum after TBI. A cytokine array using a total of 62 cytokines and chemokines (listed in table 2) was used to examine the pooled mouse serum collected 1, 3, and 6 days post-8 Gy irradiation. The unirradiated pooled serum (0 Gy) served as the baseline level. (A) Blots show duplicate measurements for each cytokine from control and three indicated time points of 8 Gy irradiated samples. Positions of G-CSF (2D), IL-10 (3E) and IL-12p^40/70^ (3F) are circled. Net light intensity images were detected using Image Gauge software. (B) The levels of cytokines and chemokines were graphed as the percentage of the positive control (representing approximately 100%) for each blot.

To verify whether there are other IL-1 family-mediated secondary cytokines in mouse serum in response to TBI, we screened release of radiation-induced cytokines in mouse serum using a commercially available mouse cytokine antibody array kit that provided antibodies for detection of 62 cytokines, chemokines, growth factors, and soluble receptors of cytokines. The majority of IL-1 family-mediated secondary cytokines were included (table 2). Pooled sera from 0 Gy control and 1, 3 and 6 day post-8 Gy irradiation groups (N = 6/group) were used for cytokine antibody array assay. Figure 4A shows array images from 0 Gy and 1, 3, and 6 days after exposure to 8 Gy, and figure 4B shows 24 cytokines and chemokines detected in mouse serum. Protein expression is shown as a density ratio normalized to positive control, and the criterion for stating a meaningful difference in protein expression between irradiated and unirradiated samples was at least a 2-fold change [32]. Interestingly, among these factors only three cytokines were changed by radiation. The level of G-CSF was enhanced whereas IL-10 and IL-12p^40/70^ (IL-12 subunit) were decreased on days 3 and 6 after radiation exposure compared with sham-irradiated control.

**Table 2:** Cytokine antibody array map.

	a	b	c	d	e	f	g	h	i	j	k	l	m	n
1	POS	POS	NEG	NEG	Blank	Axl	BLC	CD30 L	CD30	CD40	CRG-2	CTACK	CXCL 16	Eotaxin 1
2	Eotaxin 2	Fas Ligand	CX3CL1	GCSF	GM-CSF	IFN gamma	IGFBP 3	IGFBP 5	IGFBP 6	IL-1 alpha	IL-1 beta	IL-2	IL-3	IL-3 Rb
3	IL-4	IL-5	IL-6	IL-9	IL-10	IL-12 p40/70	IL-12 p70	IL-13	IL-17	KC	Leptin R	Leptin	LIX	L Selectin
4	Ltn/XCL1	MCP 1	MCP 5	M-CSF	MIG	MIP-1 alpha	MIP-1 gamma	MIP-2	MIP-3 beta	MIP-3 alpha	PF-4	P Selectin	RANTES	SCF
5	SDF-1 alpha	TARC	TCA-3	TECK	TIMP-1	TNF alpha	sTNFRI	sTNFRII	TPO	VCAM-1	VEGF	Blank	Blank	POS

BLC = B-Lymphocyte Chemoattractant; CRG-2 = Cytokine Responsive Gene-2; CTACK = Cutaneous T-cell-attracting Chemokine; CXCL = Chemokine (C-X-C motif) ligand; GCSF = Granulocyte Stimulating Factor; GM-CSF = Granulocyte-Macrophage Stimulating Factor; IFN = Interferon; IGFBP = Insulin-like Growth Factor Binding-Protein; IL = Interleukin; KC = *K*eratinocyte-derived Chemokine; LIX = lipopolysaccharide-induced CXC human chemokine; MCP = Monocyte Chemoattractant Protein; M-CSF = Macrophage Colony-Stimulating Factor; MIP = Macrophage Inflammatory Proteins; PF = Platelet Factor; RANTES = chemotactic for T cells, basophils and eosinophils; SCF = Stem Cell Factor; SDF = Stromal Cell-derived Factor; TARC = Thymus Activation Regulated Chemokine; TCA = T-cell Activation; TECK = Thymus-Expressed Chemokine; TIMP = Tissue Inhibitor of Metalloproteinases; TNF = Tumor Necrosis Factor, sTNFR = soluble Tumor Necrosis Factor Receptor; TPO = Thrombopoietin; VCAM = Vascular Cell Adhesion Molecule; VEGF = Vascular Endothelial Growth Factor; POS = Positive; NEG = Negative.

### ^60^Co TBI increased the secretion of bioactive IL-18 in circulation of rhesus macaques and Gottingen minipigs

**Figure 5:**
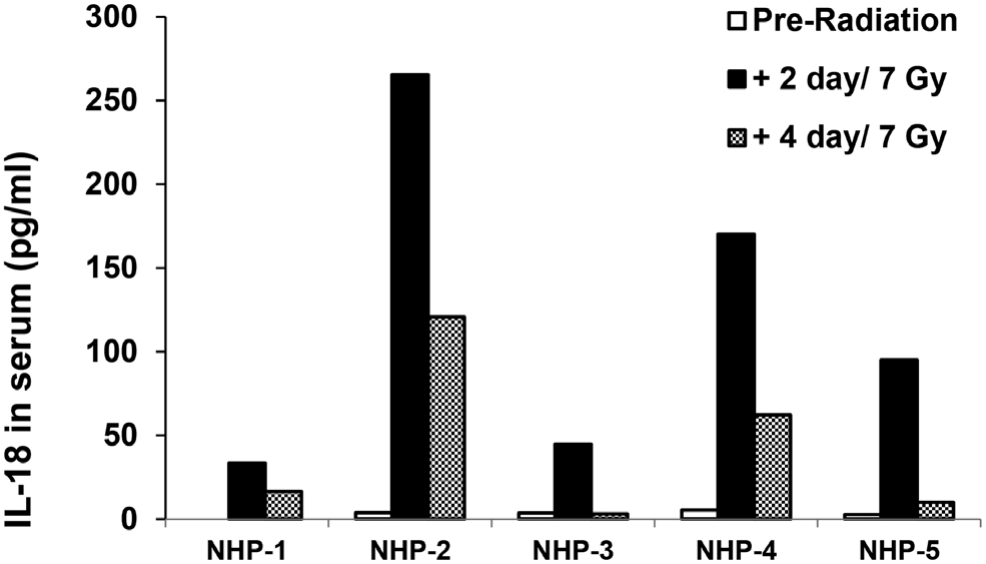
IL-18 levels in serum of Rhesus macaques in response to ^60^Co-TBI exposure. Sera were collected from five animals (3 females and 1 male) before and after 7 Gy of TBI. ELISA was performed using 100 µl of serum per individual sample. Using samples from pre-radiation as control, levels of IL-18 from same animals were evaluated at 2 and 4 days after TBI exposure. Radiation significantly increased IL-18 levels in the sera of NHPs on day 2 post-TBI, P = 0.021.

We next asked whether the elevation of circulating IL-18 also reflects radiation injury in large animal models such as NHPs and Gottingen minipigs. In this study, frozen serum from rhesus macaque and minipig plasma samples were shared with our other ongoing projects. Because the LD_50/60_ in NHPs (Rhesus macaques) is 6.44 and the LD_90/60_ is 7.56 Gy of ^60^Co γ-radiation without supportive care [8], serum samples from 5 adult rhesus macaques (4 females and 1 male, 3 to 8 years of age, and 4–8 kg of body weight) exposed to 7 Gy of ^60^Co-TBI were examined according to the methods described in “Material and Methods”. Serum samples were collected before and 2 and 4 days after 7 Gy irradiation from individual animals, and levels of IL-18 in these samples were measured using the ELISA method. Using the serum samples collected pre-radiation as control, levels of IL-18 in sera collected from the same animals 2 and 4 days after radiation were evaluated. Results in figure 5 show a significant increase of IL-18 in sera taken from all 5 animals 2 days after TBI compared to pre-irradiation samples from the same animals (p = 0.021). Four days after irradiation, radiation-induced increases of IL-18 in NHP serum were still observed in 4 (3 females and 1 male) out of 5 animals.

**Figure 6:**
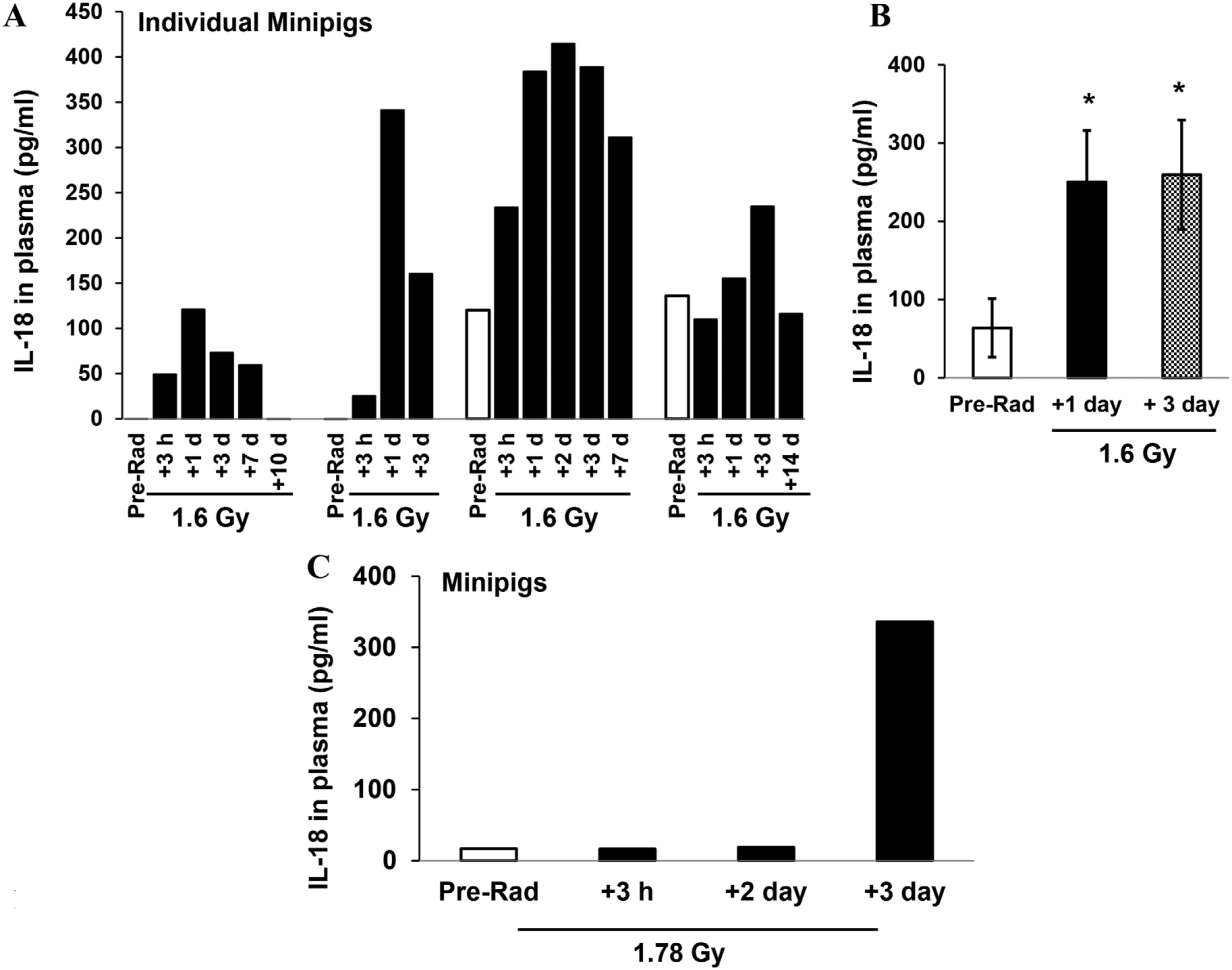
IL-18 levels in plasma of Gottingen minipigs in response to ^60^Co-TBI exposure. (A) Levels of IL-18 in plasma samples from individual animals collected before and 3 h and 1, 2, 3 and 7 days after 1.6 Gy irradiation and measured by ELISA. (B) Significant differences are shown at 1 and 3 days after 1.6 Gy. N = 4 *p<0.05; mean ± SD. (C) Levels of IL-18 were evaluated in pooled samples at different time points including pre-radiation, 3 hours, 2 days and 3 days after 1.78 Gy TBI. ELISA was performed using 100 µl of combined serum sample from each indicated time point.

We recently reported the suitability of using the Gottingen minipig as an additional large animal model for radiation research and developed dose response curves for bilateral γ-radiation of Gottingen minipigs [33]. Our results demonstrated that the Gottingen minipig is very sensitive to γ-radiation with an LD_10/30_ at 1.59 Gy, LD_50/30_ at 1.73 Gy, and LD_90/30_ at 1.86 Gy of ^60^Co-TBI, respectively [7]. Hence we decided to evaluate levels of circulating IL-18 in this animal model in response to ^60^Co-TBI. Available frozen plasma samples from male Gottingen minipigs exposed to 1.6 (N = 4) and 1.78 Gy (N = 5) of ^60^Co-TBI were examined by ELISA for quantitative detection of pig IL-18. ELISA kits with anti-minipig IL-18 antibodies that detect plasma and serum samples from the minipig were used according to methods described in “Material and Methods”. Plasma samples were collected from individual animals before and 3 h and 1, 2, 3 and 7 days after irradiation, and levels of IL-18 in these samples were measured. Using the plasma samples collected pre-irradiation as control, levels of IL-18 in the sera collected from the same animal at different time points after radiation were evaluated. Although large variations of IL-18 levels between individual animals at the same time point are shown in figure 6A, radiation significantly induced an increase of IL-18 in 1.6 Gy irradiated minipigs’ plasma at 1 and 3 days after TBI (figure 6B). Furthermore, levels of IL-18 were measured in pooled samples at each indicated time point including pre-radiation, 3 h, 2 and 3 days after 1.78 Gy of TBI due to limited sample volume. As shown in figure 6C, results indicated an extensive increase of IL-18 in the pooled minipigs’ plasma sample collected 3 days after 1.78 Gy of TBI.

### Comparison of circulating IL-18 with hematological radiation biomarkers in mice, minipigs and NHPs

**Figure 7:**
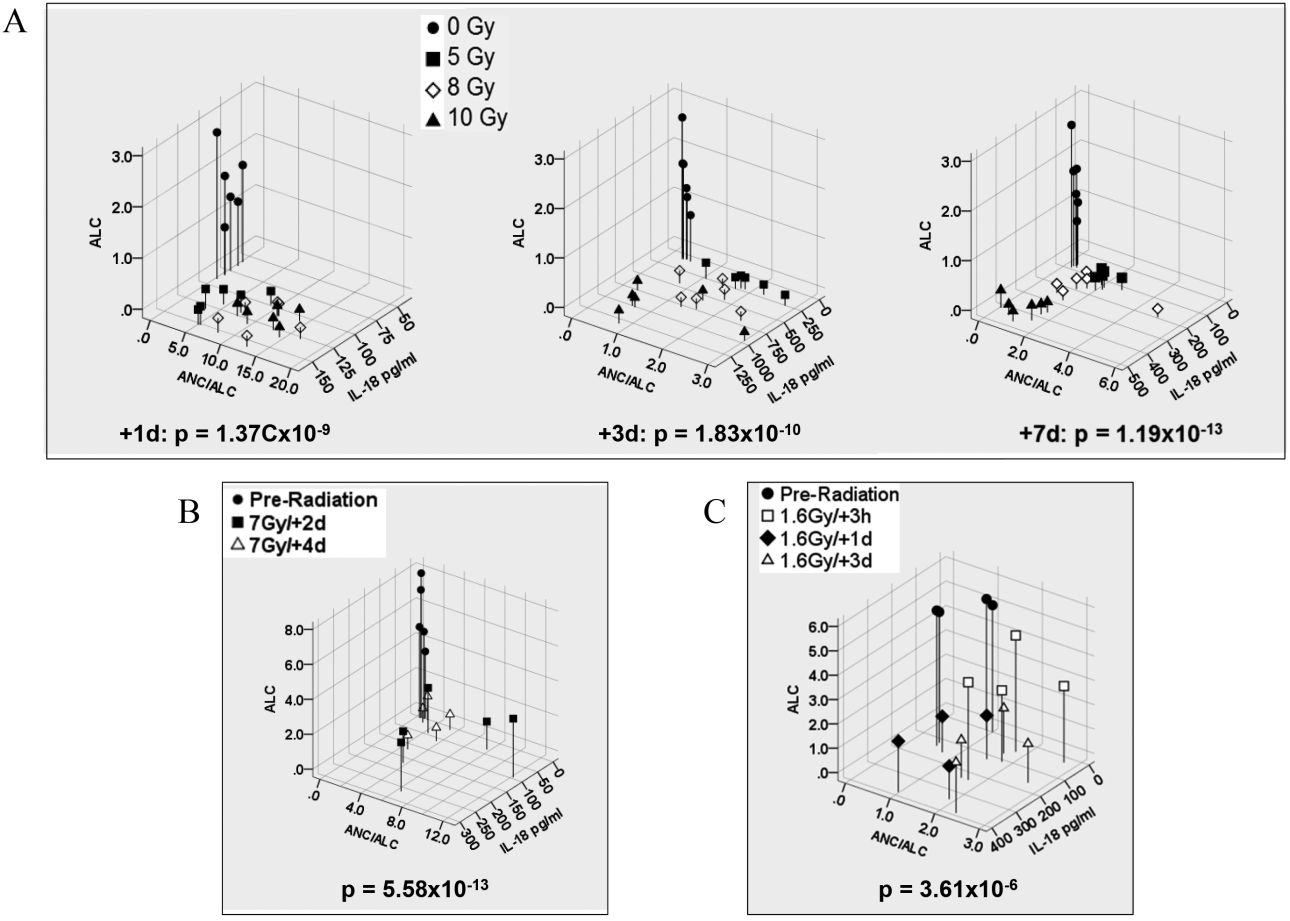
Discriminant analysis of IL-18, ALC, and ANC/ALC ratio in peripheral blood of irradiated mice, NHPs and minipigs. (A) After TBI of 0, 5, 8 and 10 Gy, the three markers from peripheral blood, IL-18, ALC, and ANC/ALC ratio were evaluated in mice at 1, 3, and 7 day post-irradiation (+1 d, +3 d, and+7 d). (B) The levels of IL-18, ALC, and ANC/ALC ratio from the same blood samples of individual NHP obtained before and after radiation (+2 days and +4 days) were compared. (C) The levels of IL-18, ALC, and ANC/ALC ratio from the same blood samples of individual minipigs obtained before and after radiation (+3 h, +1 day and +3 days) were compared. Statistical differences of three combined markers between groups were determined by multivariate analysis of variance (MANOVA). Changes in biomarker levels were significant (Wilks Lambda p<0.001) as shown in each individual panel for all combinations and time points.

Peripheral blood was collected from sham- or γ-irradiated mice, and pre- and post-irradiation in minipigs and NHPs. The absolute lymphocyte counts (ALC) and ratio of absolute neutrophil counts (ANC) to ALC (ANC/ALC) as hematology radiation biomarkers [29] were compared with circulating IL-18 from same samples of unirradiated and irradiated animals. The discrimination of radiation-induced IL-18, ALC and ANC/ALC in mice, NHPs and minipigs were analyzed and results are shown in figure 7. Mouse blood samples were collected on 1, 3, and 7 days after 0, 5, 8, or 10 Gy. A significant reduction of ALC and corresponding IL-18 increase were observed in a radiation dose-dependent fashion compared with sham–irradiated samples (p<0.01) (figure 7A). ANC levels were increased on day 1, followed by significant decreases on day 3 and 7 after radiation, resulting in an elevation of ANC/ALC ratio only at 24 h post-radiation. Consistent with the mouse study, radiation-induced ALC decreases and IL-18 increases were observed at all tested time points in NHPs (figure 7B) and minipigs (figure 7C). The ANC/ALC ratio was increased 2 and 4 days after 7 Gy in NHPs and 3 h and 1 and 3 days after 1.6 Gy in minipigs. However, ratios of ANC/ALC were found overlapping in after different radiation doses irradiated animals, suggesting variability of this measure in response to γ-radiation.

## Discussion

Ionizing radiation can induce a variety of biological injuries depending on the physical nature, duration, doses and dose-rates of exposure [2], [34]. Information from individual exposures is essential for early triage during radiological incidents to provide optimum possible life-sparing medical procedures to each person [35]. A rapid, easy-to-use, inexpensive and accurate radiation dose-assessment assay that tests easily obtained samples such as blood or urine with transportable equipment is in urgent need in emergency scenarios to triage and track radiological casualties, and to evaluate the radioprotective and mitigative/therapeutic effects of radiation countermeasures [10], [11], [36]. A good biomarker should be specific in differentiating pathologies, sensitive to facilitate rapid and significant detection before or during the development of pathology, and stable in different conditions so it can be extracted from biopsies fixed for diagnostic staining or from stored body fluids. However, this biomarker is not yet available.

Whole-body radiation-induced multi-tissue injury could result in specific antigen secretion. Ionizing radiation-induced inflammatory cytokine and chemokine production and secretion from injured cells may result in stress response signal activation and cell damage and depletion [12], [15], [37]. The IL-1 family of cytokines plays key roles in inflammatory and immune responses [21], therefore it can be the first line of host defense against stresses [19], [20]. In the current study, we examined the effects of γ-radiation on three IL-1 family cytokines, IL-1β, IL-18, and IL-33. We also studied their secondary cytokines’ expression and secretion in different mouse tissues and serum using ELISA, immunoblotting and mouse cytokine antibody array as these cytokines have been described as significantly influencing pathogenesis of diseases, including radiation injury [12], [21], [38], [39]. We expected to identify biomarkers that can be used easily as accurate radiation dose-assessment assays after acute radiation injury. Our data identified significant increases of IL-1β, IL-18, and/or IL-33 in mouse thymus, spleen and BM cells after irradiation. However, levels of these cytokines varied in different tissues in response to the same dose of radiation at indicated time points, and it is difficult to determine which of these cytokines is the best radiation biomarker candidate. We further examined these cytokines in mouse serum and hypothesized that elevated levels of circulating IL-1 family cytokines in serum after total-body radiation may reflect proportionally the radiation doses and severity of radiation injury in individual animals. Interestingly, of all cytokines we examined, only IL-18 increased significantly and persisted in mouse serum for at least 13 days after irradiation (figure 3). In four independent experiments with 6 mice per group in each experiment (total N = 24/group), radiation-induced IL-18 increases in mouse serum were observed on day 1 post-irradiation and continually increased and reached a peak on day 3 with 4.5 to 24-fold increases after 5–12 Gy compared to serum samples from sham-irradiated controls. The sensitivity and specificity of circulating IL-18 increases in response to gamma radiation were evaluated by receiver operator characteristic (ROC) curves with 95% confidence intervals (CI), which is a recommended standard statistical method for development of biomarkers [31]. Furthermore, we found that serum samples undergoing several freeze and thaw cycles produced almost identical readings in ELISA, suggesting flexibility in storage conditions.

To verify whether there are other cytokines in mouse serum in response to TBI, we screened release of radiation-induced cytokines in mouse serum using a mouse cytokine antibody array kit including 62 cytokines, chemokines, growth factors, and soluble receptors of cytokines. The majority of IL-1 family-mediated secondary cytokines were included. Three cytokines responded to radiation with one showing an increase (G-CSF) and two a decrease (IL-10 and IL-12p^40/70^). G-CSF is a secondary cytokine of IL-1β [24], and its increase may have reflected IL-1β activation in radiation injury. However, radiation-induced G-CSF increases happened relatively late (3 days post-radiation) and the level of G-CSF in irradiated mouse serum was low, as shown in figure 4. Although it was detected by cytokine antibody array, the result may not be detectable by ELISA. Thus, G-CSF may not be as good a radiation biomarker as IL-18.

We further confirmed our finding in frozen minipig plasma and NHP serum after sham- and γ-irradiation and demonstrated that radiation significantly enhanced IL-18 in serum from 5 NHPs 2 to 4 days after 7 Gy irradiation and plasma from minipigs (samples of total 9 animals) 1–3 days after 1.6 and 1.78 Gy irradiation. Because hematological biomarkers of exposure to ionizing radiation are well characterized and used in medical management of radiological casualties [28], in this study, comparisons were also made between the new radiation biomarker IL-18 and the well-known hematological radiation biomarkers [29] in three animal models. Our data demonstrated that a significant reduction of absolute lymphocyte counts (ALC) in animal blood after radiation was negatively correlated with radiation-induced increases in circulating IL-18. Thus, for the first time we demonstrated that circulating IL-18 increased and existed stably in mice, minipigs and NHPs after ^60^Co γ- irradiation in a radiation dose and time-dependent manner, although the radiation-tolerance levels in these animals differ significantly.

Recent studies suggested that IL-33 represents a group of IL-1 family factors which retain some intracellular functions and are passively externalized upon cell lysis. In contrast, IL-1β and IL-18 are induced in restricted inflammatory cells by inflammatory stimuli and undergo regulated secretion [20], [40]. Interestingly, there are specific differences between IL-1β and IL-18. For example, an IL-18 precursor is present constitutively in almost all cells including hematopoietic cells, mesenchymal cells, and epithelial cells of the GI tract in healthy humans and animals, whereas the IL-1β precursor rarely is found in these cells [23]. IL-1β is produced by monocytes, macrophages, dendritic cells (DC), B-lymphocytes and nature killer (NK) cells [41]. In addition, IL-1β activation of cells usually needs picograms (pg) to nanograms (ng) per milliliter (mL), whereas IL-18 requires 10–20 ng/mL or even more [42]. Consistently, our results demonstrated that IL-18 was increased significantly and was easily measured using the ELISA method in mouse and NHP serum and minipig plasma after TBI, and levels of IL-18 in the sera of the three animal models were correlated tightly with radiation exposure. In contrast, IL-1β in mouse serum was undetectable by ELISA regardless of radiation. The results comprise a proof of concept that measurement of IL-18 in blood may be useful for estimating radiation exposure at the indicated time points. Furthermore, using the inexpensive and easy-to-use ELISA method to evaluate the level of IL-18 from easily obtained serum samples after radiation exposure may be useful to assess the activity and severity of radiation-induced damage, and to track health status after radiation injury and therapy. The mechanism(s) by which radiation regulates inflammatory cytokine IL-18 expression and secretion are under investigation.

Shan at al. recently demonstrated that whole-body low dose (0.05 and 0.075 Gy) ionizing radiation (X-ray) induced IL-12 and IL-18 secretion by mouse peritoneal macrophages [13]. Another report by Kang et al. indicated that low dose (10 cGy) of TBI or half-body radiation (only the area below the xyphoid process was irradiated) upregulated IL-18 mRNA expression in mouse peripheral blood mononuclear cells [43]. It suggests that low dose radiation promotes the innate immune response which can induce IL-18 expression. In future studies, we will evaluate circulating IL-18 after low dose (<100 mSv or 1 Gy) gamma radiation in mouse and/or large animal models, since radiation accidents can cause low dose radiation exposure and low dose radiation-induced health risks not only involve neoplastic diseases but also mutations that may contribute to different diseases [44].

## Materials and Methods

### Ethics Statement

Animals were housed in an Association for Assessment and Accreditation of Laboratory Animal Care (AAALAC) -approved facility at the Armed Forces Radiobiology Research Institute (AFRRI). All procedures involving animals were reviewed and approved by the AFRRI Institutional Animal Care and Use Committee (IACUC) and all efforts were made to minimize suffering. Animals received total-body irradiation (TBI) in a bilateral gamma radiation field at AFRRI’s Cobalt-60 (^60^Co) facility. The day of irradiation was considered day 0. Control animals were sham-irradiated and treated in the same manner as the irradiated animals, except the ^60^Co source was not raised from the shielding water pool.

### Animals

#### Mice

Twelve- to 14-week-old CD2F1 male mice (Harlan Laboratories, Indianapolis, IN) were used according to methods described in previous reports [30]. All animals were acclimatized upon arrival and representative animals were screened for evidence of disease. Animal rooms were maintained at 21±2°C with 50%±10% humidity on a 12 h light/dark cycle. Commercial rodent chow (Harlan Teklad Rodent Diet 8604) was available ad libitum as was acidified water (pH ¼ 2.5) to control opportunistic infections. Animals were chosen randomly for each experimental group and received either 0 (sham-irradiation), 5, 7, 8, 9, 10 or 12 Gy at a dose rate of 0.6 Gy/min. After irradiation, mice were returned to their home cages with food and water provided as usual.

#### Minipigs

Male Gottingen minipigs (Total 9 animals were used in this study. 4 months of age, 9–11 kg) were obtained from Marshall BioResources (North Rose, NY). The Gottingen minipig is the smallest minipig available specifically bred for biomedical purposes. Procedures were performed in accordance with protocols approved by the AFRRI-IACUC as previously reported [33]. Briefly, minipigs were singly housed in adjoining cages that allowed tactile, visual, olfactory and auditory contact through cage bars. Room temperature was kept between 64 and 79°F (17.8 to 26.1°C) and humidity between 50%±20%. Environmental enrichment and stimulation were provided in the form of physical devices (treats, sanitized toys) and positive interactions with caretakers. Minipigs were fed twice daily (Harlan Teklad Minipig diet 8753, Madison, WI, USA) according to individual weights and provider recommendations; water was provided ad libitum. The animals were subjected to total bilateral body irradiation using ^60^Co, with radiation doses of 1.6 and 1.78 Gy at a dose rate of 0.6 Gy/min as described in our previous report. After irradiation, minipigs were returned to their home cages with food and water provided as usual.

#### Nonhuman primates (NHPs)

Rhesus macaques (4 female and 1 male, 3 to 8 years of age, and 4–8 kg of body weight) used in the present study was part of an ongoing project on evaluation of radiation countermeasures in NHPs. Research with animals was conducted according to the principles enunciated in the Guide for the Care and Use of Laboratory Animals prepared by the Institute of Laboratory Animal Resources, National Research Council. The animal protocol describing care, radiation and blood collection was approved by the AFRRI-IACUC and the radiation procedure has been described previously [8], [35]. Briefly, Rhesus macaques were housed individually in sanitized stainless-steel cages in conventional holding rooms provided with a minimum of 10–15 changes/h of 100% fresh air, conditioned to 18–29°C and a relative humidity of 50%±20% on a 6∶00 o’clock light–18∶00 o’clock dark full-spectrum light cycle. Environmental enrichment and stimulation were provided in the form of physical devices (treats, sanitized toys) and positive interactions with caretakers. Animal were fed twice daily (Teklad Global 20% Protein Primate Diet Jumbo T-2050J) according to individual weights and provider recommendations. Diets were supplemented with fruit, vegetables and liquid diets, and water was provided ad libitum. Ketamine-anesthetized Rhesus macaques were placed in Plexiglas chairs and exposed to a total-body radiation to midline tissue dose of 7.0 Gy at dose rate of 0.6 Gy/min. After irradiation, animal were returned to their home cages with food and water provided as usual.

### Mouse peripheral blood cell counts and serum and tissue preparation

On days 1, 3, 6, 9 and 13 after TBI, mice were humanely euthanized for serum and tissue collection. Euthanasia was carried out in accordance with the recommendations and guidelines of the American Veterinary Medical Association. The mice were deeply anesthetized prior to collecting whole blood through a cardiac blood draw in accordance with the approved IACUC protocol. The blood was immediately divided into two tubes. The samples in EDTA tubes were used for peripheral blood cell counts by a clinical hematology analyzer (Bayer Advia 120, Bayer, Tarrytown, NY) at the AFRRI Veterinary Sciences Department facility, and samples in BD Microtainer Gold tubes were left unmoved on racks. Following 30 minute coagulation at RT, the sera were well separated from the gel by 10 minute-centrifugation at 10,000×g/min, collected and stored at –80°C for later study. Once blood collection from individual mice and the mouse euthanasia were completed, mouse tissues were collected. Bone marrow cells were collected from mouse femurs and humeri. After erythrocytes were lysed by erythrocyte lysis buffer (Qiagen GmbH, Hilden, USA), total bone marrow myeloid cells were collected for further experiment use. Mouse spleens and thymuses were excised, rinsed with PBS, and snap-frozen in liquid nitrogen then stored at –80°C for further use.

### Protein extraction and immunoblotting

The frozen mouse tissues (spleens, thymuses, livers, and lungs) were homogenized in 1X radio-immunoprecipitation assay buffer (RIPA, Sigma-Aldrich, St Louis, MO, USA) (supplemented with a protease inhibitor tablet) by tissue homogenizer (Fast Prep-24, MP Biomedicals, Solon, OH, USA), following the manufacturer’s recommendations. After 15-min centrifugation at 12,000×g/min, the supernatant was collected and protein concentrations were determined using a BCA assay kit (Pierce, Rockford, IL, USA). The collected homogenates were denatured in Laemmli buffer supplemented with DTT (dithiothreitol), and the same amount of protein from each sample (100 to 120 µg) was loaded for SDS-PAGE electrophoresis. Subsequently, immunoblotting was performed following standard procedures with an enhanced chemiluminescence kit (Thermo Scientific, Rockford, IL, USA). The images were captured by CCD camera and the resulting densitometry was assessed using ImageGauge software. Protein densitometry was normalized to beta-actin. Antibodies for mouse IL-1β and IL-18 were purchased from R&D (Minneapolis, MN, USA), for beta-actin from Sigma-Aldrich (St Louis, MO, USA), for mouse IL-33 from Santa Cruz (Santa Cruz Biotechnology, Dallas, TX, USA).

### Blood sampling from minipigs and NHPs

To facilitate collection of minipig blood samples according to the IACUC protocol, animals were quarantined for two weeks and implanted with a vascular access port (VAP). After 3 weeks of recovery from surgical implantation of the VAP, their blood samples were obtained from the VAP before and after irradiation at indicated time points. Blood was collected via a strictly aseptic technique in sample tubes containing EDTA and immediately stored on ice until further processing. After blood sample collection, the animals were returned to their original cages.

NHP blood samples from individual animals were collected for all experimental time points (pre-irradiation, 2, and 4 day post-irradiation) according to the IACUC protocol. Blood was collected from a peripheral vessel or femoral vein with a 22–25 G heparinized needle/syringe. Serum samples were maintained at –70°C until assay. After blood sample collection, the animals were returned to their original cages.

Both minipigs and NHPs’ peripheral blood cell counts were performed at the AFRRI Veterinary Sciences Department facility using a clinical hematology analyzer (Bayer Advia 120, Bayer, Tarrytown, NY).

### Cytokine quantitation by enzyme-linked immune sorbent assay (ELISA)

Quantitation of IL-1β, IL-6, IL-8, IL-18, and IL-33 was performed using ELISA kits suitable for detecting these cytokines in sera and cell lysates. Cytokine levels in BM cells, spleen and thymus tissue homogenate were determined following assay instructions provided by manufacturers. Briefly, BM cells after erythrocyte removal, spleen and thymus tissue from individual mice were homogenized and sonicated in PBS plus proteinase inhibitor, followed by 15 min of 12,000×g centrifugation. The supernatant was collected and subjected to protein determination (BCA assay). The supernatant with an equivalent amount of protein (10 to 100 µg) from each sample was evaluated in duplicate. Statistical analysis was conducted from group samples of 6 mice.

ELISA kits for determining cytokines in mouse serum and tissues were purchased from R&D (Minneapolis, MN, USA). The IL-18 ELISA kits for minipigs and NHPs were purchased from Bioscience (San Diego, CA, USA). The limits of detection of IL-18 are 25 and 23 pg/ml for mouse and minipig samples, respectively.

### Cytokine antibody array

Pooled mouse sera from 6 mice/group were subjected to cytokine antibody array analysis using the Ray Bio Mouse Cytokine Antibody Array 3 kit (Ray Biotech, Inc. Norcross, GA, USA) according to manufacturer’s instructions. In brief, the array membrane coated with cytokine antibodies was first blocked with blocking buffer and then incubated with 1.0 mL of pooled mouse sera (1∶3 dilution) followed by washing and incubation with biotin-conjugated second antibody and horseradish peroxidase-conjugated streptavidin. The membrane was developed using enhanced chemiluminescence solution (Thermo Scientific, Rockford, IL, USA) and exposed to x-ray film. Protein expression was expressed as a percentage density normalized to background and calculated using Fuji SuperArray analysis.

### Statistical analysis

Differences between means were compared by one way analysis of variance (ANOVA) with Dunnett’s Post-Hoc test and multivariate analysis of variance (MANOVA). P<0.05 was considered statistically significant. Results are presented as means ± standard deviations or standard errors of the mean as indicated. The sensitivity and specificity of single biomarker were analyzed by the receiver operator characteristic (ROC) curve using IBM-SPSS program (SPSS Statistics Professional). Results are presented as area under ROC curves (AUC) with 95% confidence interval (CI).
